# High Glucose-Induced ROS Production Stimulates Proliferation of Pancreatic Cancer via Inactivating the JNK Pathway

**DOI:** 10.1155/2018/6917206

**Published:** 2018-11-21

**Authors:** Jiao Luo, Yukai Xiang, Xiangxiang Xu, Dazhang Fang, Ding Li, Fubiao Ni, Xiandong Zhu, Bicheng Chen, Mengtao Zhou

**Affiliations:** ^1^Department of Surgery, The First Affiliated Hospital of Wenzhou Medical University, Wenzhou, 325000 Zhejiang Province, China; ^2^Key Laboratory of Diagnosis and Treatment of Severe Hepato-Pancreatic Diseases of Zhejiang Province, The First Affiliated Hospital of Wenzhou Medical University, Wenzhou, 325000 Zhejiang Province, China

## Abstract

Aberrant glucose metabolism of diabetes mellitus or hyperglycemia stimulates pancreatic tumorigenesis and progression. Hyperglycemic environment can increase the ROS level of tumors, but the role of upregulation of ROS levels in pancreatic cancer (PC) still remains controversial. Here, the same as other reports, we demonstrate that high glucose promoted pancreatic cancer cell growth and resulted in an increase in the level of ROS. However, it is interesting that the phosphorylation of JNK was reduced. When treating PC cells with N-acetyl-L-cysteine (NAC), the intracellular ROS generation is repressed, but the expression of phosphorylation of JNK and c-Jun increased. Moreover, the JNK inhibitor SP600125 significantly promoted cell proliferation and suppressed cell apoptosis of pancreatic cancer cells under high glucose conditions. Collectively, high levels of ROS induced by high glucose conditions stimulated the proliferation of pancreatic cancer cells, and it may be achieved by inactivating the JNK pathway.

## 1. Introduction

As one among the most fatal malignancies, pancreatic ductal adenocarcinoma (PDAC) has the record of lower than 5% survival rate within five years. PDAC is usually asymptomatic at the early stage and diagnosed in an advanced stage [1–3]. Parallel to an incidence on the rise, the surge in obesity and metabolic syndrome is also a risk factor for PDAC [4–6]. Diabetes mellitus (DM) as a complex disease characterized by hyperglycemia may play a critical role facilitating the progression and metastasis of several types of cancer [7, 8]. When considering the relationship of diabetes as an early manifestation and an independent risk factor for PDAC, the underlying mechanisms whether and by which way high glucose (HG) stimulates pancreatic tumorigenesis remain mostly unclear [9].

Reactive oxygen species (ROS) are generally considered as by-products of oxygen consumption and cellular metabolism. It has been demonstrated previously that high glucose can influence pancreatic tumorigenesis and progression through oxidative stress [10]. Most pancreatic cancer patients suffer with diabetes or hyperglycemia, and high glucose can promote the production of ROS which may be related to the enhanced invasion and migration activity of pancreatic cancer cells [11], whereas the influence of ROS on the proliferation of pancreatic cancer still remains controversial. In fact, cellular ROS generation can be considered as a double-edged sword. Excess ROS production can cause damage to DNA, proteins, and lipids; interfere with cellular signaling pathways; and induce apoptosis or necroptosis [12–14]; a moderate increase in ROS may have a mitogenic effect in tumors, maintain redox equilibrium, and promote cell proliferation as well [15, 16].

As a member of the MAPK family, c-Jun-N-terminal kinase (JNK) is an important signal transduction pathway for regulating cell proliferation, differentiation, and apoptosis [17, 18]. Being an important signaling cascade downstream of ROS, the roles of JNK from various perspectives of tumorigenesis and tumor progression containing cancer stem cell maintenance are gradually being realized now [19]. It has also been reported that ROS-mediated cellular damage is closely associated with persistent activation of the JNK pathway [20]. It seems that the persistent activation of JNK boosts cell death by mitochondrial ROS or by interfacing with the components of the intrinsic apoptotic pathway [21]. Moreover, in hepatocellular carcinoma, high glucose supports cell proliferation and in vivo tumor growth and inhibits apoptosis by suppressing the activation of the JNK pathway [22]. But the effects of JNK are not clear on pancreatic cancer under high glucose conditions.

In this study, we demonstrate that high glucose is capable of promoting pancreatic cancer cell proliferation and increasing the level of ROS. However, in contrast to other reports [18, 23], the activity of JNK was inhibited by the increase in ROS levels. We hypothesize that high levels of ROS induced by high glucose conditions stimulates the proliferation of pancreatic cancer cells, and it may be achieved by inactivating the JNK pathway.

## 2. Materials and Methods

### 2.1. Materials

Fetal bovine serum (FBS), Dulbecco's modified Eagle's medium (DMEM), and trypsin were purchased from Gibco Life Technologies (Grand Island, NY, USA). D-Glucose (G8270) and N-acetyl cysteine (NAC; A7250) were purchased from Sigma Chemical (St. Louis, MO, USA). The JNK inhibitor SP600125 was purchased from Selleck Chemicals (s1460; Houston, TX, USA). The antibodies used in this study were against CDK2 (ab32147), active caspase-3 (ab2302), and Ki-67 (ab16667; Abcam Inc., MA, USA) and GAPDH (5174S), JNK (9252T), phospho-JNK (4668T), c-Jun (9165P), phospho-c-Jun (3270P), cyclin D1 (2978), p21 (2947), Bax (2772S), and Bcl-2 (15071S; Cell Signaling Technology Inc., MA, USA).

### 2.2. Cell Culture

In this experiment, we used the human pancreatic cancer cell lines, PANC-1 and CFPAC-1. The cells were purchased from the Cell Bank of the Chinese Academy of Sciences (Shanghai, China) and were cultured in low-glucose (5 mM; LG) DMEM supplemented with 10% FBS, 100 U/mL penicillin, and 100 *μ*g/mL streptomycin. Cells were incubated at 37°C atmosphere containing 5% CO_2_ and passaged using 0.25% trypsin-EDTA solution. Cells were exposed to high-glucose (25 mM; HG) DMEM for 24 h to study the effects of glucose concentration on them. In addition, 10 *μ*M SP600125 or 5 mM NAC was added to the culture medium.

### 2.3. Colony Formation Assay

3 × 10^3^ cells per well were seeded into a 12-well plate incubated in media containing several concentrations of glucose and treated with or without 5 mM NAC. After one week, the cells grew to visible colonies. The colonies were washed once with PBS and fixed using 4% paraformaldehyde for 20 min. Then, cells were stained with 0.1% crystal violet for 15 min, and the number of colonies per well was counted by ImageJ. All *P* values were calculated using one-way ANOVA.

### 2.4. Cell Cycle Analysis

1 × 10^5^ cells were seeded in a 6-well plate. After being cultured in media containing 25 mM glucose with or without NAC (5 mM) for 24 h, cells were collected and washed with phosphate-buffered saline (PBS) twice and fixed with 70% ethanol overnight at −20°C. Then, cells were stained with 10 *μ*L propidium iodide (PI; MultiSciences, Shanghai, China) for 30 min in the dark. Fluorescence intensity was analyzed using a flow cytometry (BD Biosciences, USA), and the data was analyzed by FlowJo 7.6.

### 2.5. Detection of Intracellular ROS

Intracellular ROS generation was measured by a reactive oxygen species assay kit (E004; Nanjing Jiancheng Bioengineering Institute, Nanjing, China) using DCFH-DA as the molecular probe. 1 × 10^5^ cells were seeded in a 6-well plate overnight and then cultured in HG media with or without NAC (5 mM) for 24 h. Subsequently, cells were stained with DCFH-DA (10 *μ*M) for 30 min in the dark and washed twice with PBS. Cells were collected, and fluorescence was detected with a flow cytometry (BD Biosciences, USA). The data was analyzed by FlowJo 7.6.

### 2.6. Immunofluorescence Analysis

Immunofluorescence was used to detect the influence of high glucose on the expression of Ki67. After treatment with high glucose for 1, 6, and 24 h, respectively, cells were washed with PBS, fixed with 4% paraformaldehyde for 15 min at 4°C, and then permeated with 0.5% Triton X-100 for 10 min. After blocking with 5% BSA for 1 h, the cells were incubated with Ki67 (1 : 200) antibody overnight at 4°C. Following, the slides were incubated for 1 h at 37°C with DyLight 594-conjugated goat anti-rabbit IgG antibody (BioSharp, Technology Inc., China). The nuclei were then stained with DAPI solution. Pictures were taken by immunofluorescence microscopy (Leica Microsystems, CMS GmbH, Wetzlar, Germany).

### 2.7. Western Blot Analyses

After treatments, cells were lysed in a radioimmunoprecipitation assay buffer (P0013B; Beyotime Biotechnology, Jiangsu, China) containing 1% phenylmethylsulfonyl fluoride (ST506; Beyotime Biotechnology, Jiangsu, China) and 10% phosphatase inhibitor (5892791001; Roche Diagnostics GmbH, Mannheim, Germany) for 30 minutes. Then, the cell lysate was centrifuged at 12,000 × g for 15 min, and the supernatant was collected. Protein concentrations were determined using the BCA assay kit (P0012; Beyotime Biotechnology, Jiangsu, China). The proteins were separated using sodium dodecyl sulfate polyacrylamide gel electrophoresis (SDS-PAGE) and then transferred to polyvinylidene fluoride (PVDF) membranes (Millipore Corporation, Bedford, MA, USA). The blots were incubated with primary antibodies at 4°C overnight and then with a horseradish peroxidase- (HRP-) conjugated goat anti-rabbit IgG antibody (BioSharp, Technology Inc., China) diluted to 1 : 10000 at room temperature for 1 h. Primary antibodies to the following proteins were used: Bax (1 : 1000), Bcl-2 (1 : 1000), JNK (1 : 1000), p-JNK (1 : 1000), c-Jun (1 : 1000), p-c-Jun (1 : 1000), cyclin D1 (1 : 1000), p21 (1 : 1000), CDK2 (1 : 1000), and GAPDH (1 : 10000). Protein bands were detected using Image Lab Software (Bio-Rad Laboratories Inc., Berkeley, CA, USA).

### 2.8. Statistical Analyses

All data are given as mean ± SD and analyzed by one-way ANOVA. Statistical significance was determined as *P* < 0.05. All data were obtained from independent experiments over three times.

## 3. Result

### 3.1. High Glucose Promoted Pancreatic Cancer Cell Proliferation

**Figure 1:**
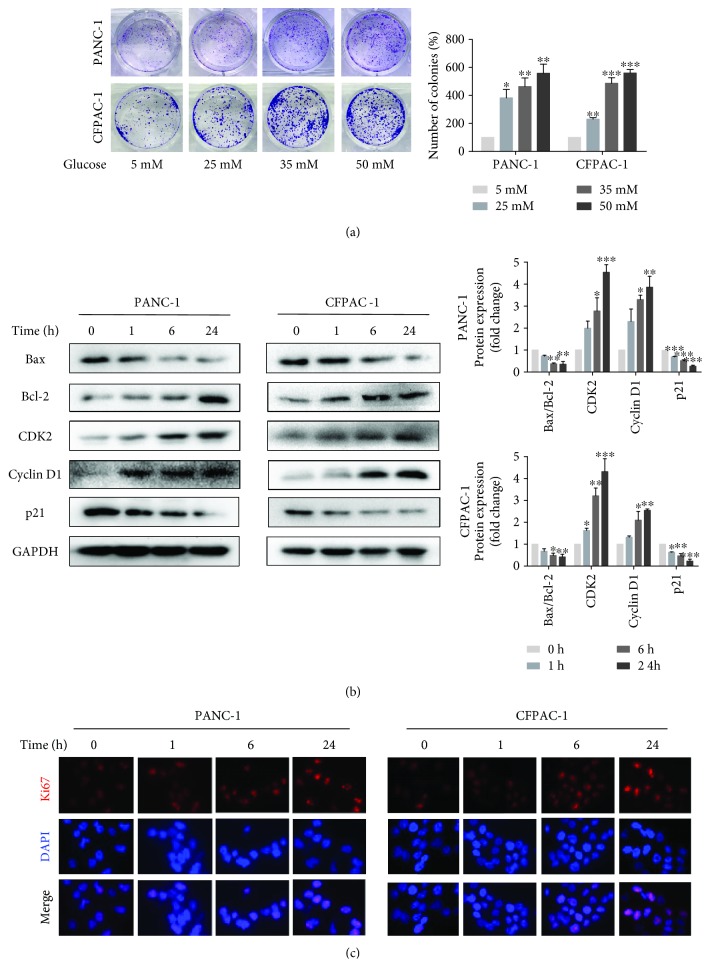
High glucose promotes pancreatic cancer cell proliferation. (a) Cell viability was measured by colony formation assay after exposure to various concentrations of glucose that range from 5 mM to 50 mM. (b) Western blotting results of Bax, Bcl-2, CDK2, cyclin D1, and p21 in PANC-1 and CFPAC-1 cells after incubating in HG media for 0, 1, 6, or 24 h. (c) The fluorescence intensity of Ki67 (red) after culturing with HG media for 0, 1, 6, and 24 h was detected using immunofluorescence microscopy (×400). Cell nuclei were counterstained with DAPI (blue). Data are presented as mean ± standard error of the mean (SEM) (*n* = 3). ^∗^*p* < 0.05, ^∗∗^*p* < 0.01, and ^∗∗∗^*p* < 0.001, compared with the 0 h group.

Firstly, we examined the effect of glucose on PC cell proliferation. The colony formation assay revealed that high glucose causes a significant increase in the proliferative capability of the tumor cells in a concentration-dependent manner (Figure 1(a)). Then, we detected the influence of high-glucose stimulation on PC cell proliferation at different times by Western blot analysis. As shown in Figure 1(b), with stimulation time increasing, the expression of cyclin D1 and CDK2 increased, while the ratio of Bax/Bcl-2 and the expression of p21 decreased. In addition, we explored the expression of Ki67 which was well-known as a marker for cellular proliferation by immunofluorescence analysis. The Ki67 proteins were more abundant in nuclei of high glucose-treated PC cells than in nontreated cells, and fluorescence intensity increases with the addition of the stimulation time of high glucose (Figure 1(c)). The above results showed that high glucose indeed stimulated PC cell proliferation.

### 3.2. The Alteration of ROS and JNK-Mediated Signaling Pathway in HG Conditions

**Figure 2:**
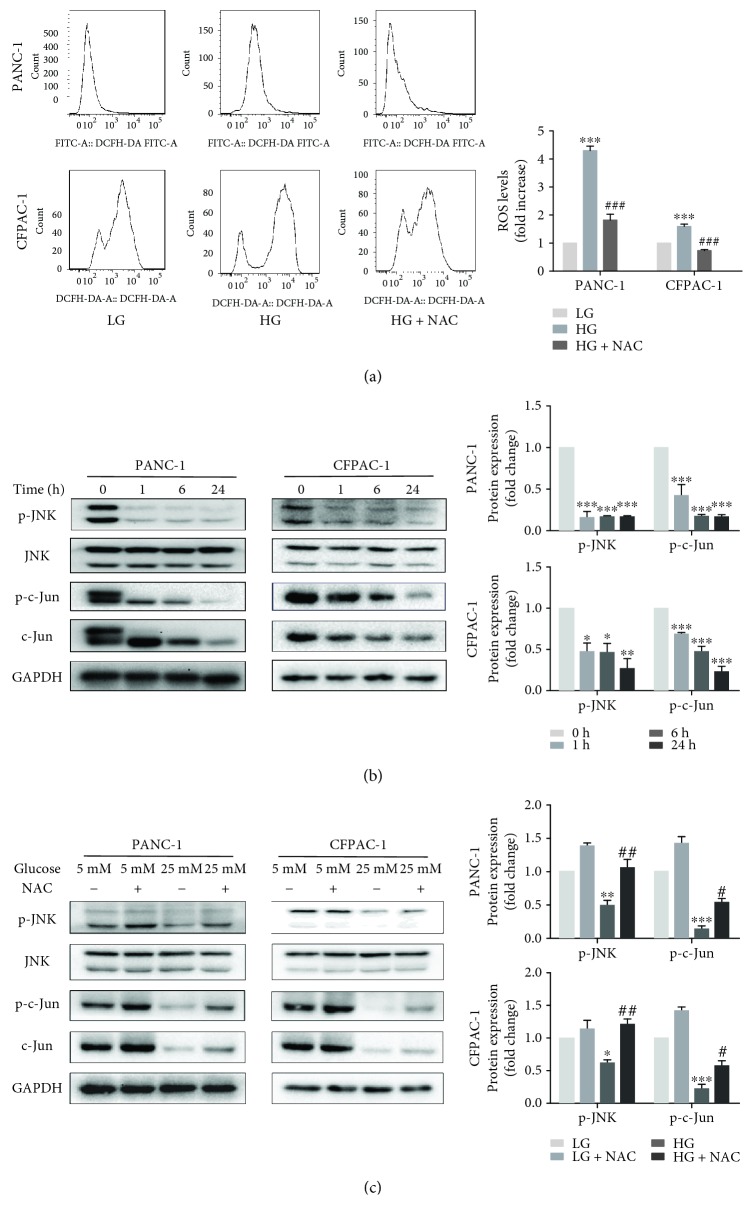
The alteration of the ROS and JNK-mediated signaling pathway in HG conditions. (a) Following treatment of PANC-1 and CFPAC-1 cells with LG, HG, and HG with NAC (5 mM) for 24 h, ROS levels were measured using the ROS fluorescent probe DCFH-DA with flow cytometry. (b) Following treatment with HG for 0, 1, 6, or 24 h, the protein expression levels of p-JNK, JNK, p-c-Jun, c-Jun, and GAPDH were determined by Western blotting in PANC-1 and CFPAC-1 cells. (c) PANC-1 and CFPAC-1 cells were treated with LG and HG for 24 h in the presence or absence of NAC (5 mM). The protein expression levels of p-JNK, JNK, p-c-Jun, c-Jun, and GAPDH were determined by Western blotting. Data are presented as mean ± SEM (*n* = 3). ^∗^*p* < 0.05, ^∗∗^*p* < 0.01, and ^∗∗∗^*p* < 0.001, compared with the LG group. ^#^*p* < 0.05, ^##^*p* < 0.01, and ^###^*p* < 0.001, compared with the HG group.

Following, we demonstrated that high glucose significantly increased the level of ROS (Figure 2(a)) using the ROS fluorescent probe DCFH-DA to detect changes in intracellular ROS and analyzing by subsequent flow cytometry. However, PC cells were exposed to high-glucose media and incubated with NAC (5 mmol/L) for 24 h, and ROS levels were decreased obviously (Figure 2(a)). In addition, to determine if high glucose affects JNK signaling, phosphorylated levels of JNK and c-Jun were evaluated by Western blot analysis. We discovered that p-JNK and p-c-Jun levels were continuously reduced in a time-dependent manner in HG conditions (Figure 2(b)). Furthermore, through Western blot analysis, we learnt that the ROS scavenging agent (NAC) enhanced JNK activation in HG-treated PC cells (Figure 2(c)). These data revealed that ROS might possibly work as an upstream effector of the JNK-mediated signaling pathway during HG-induced proliferation of PC cells.

### 3.3. Suppression of ROS Activation Would Reduce Cell Proliferation

**Figure 3:**
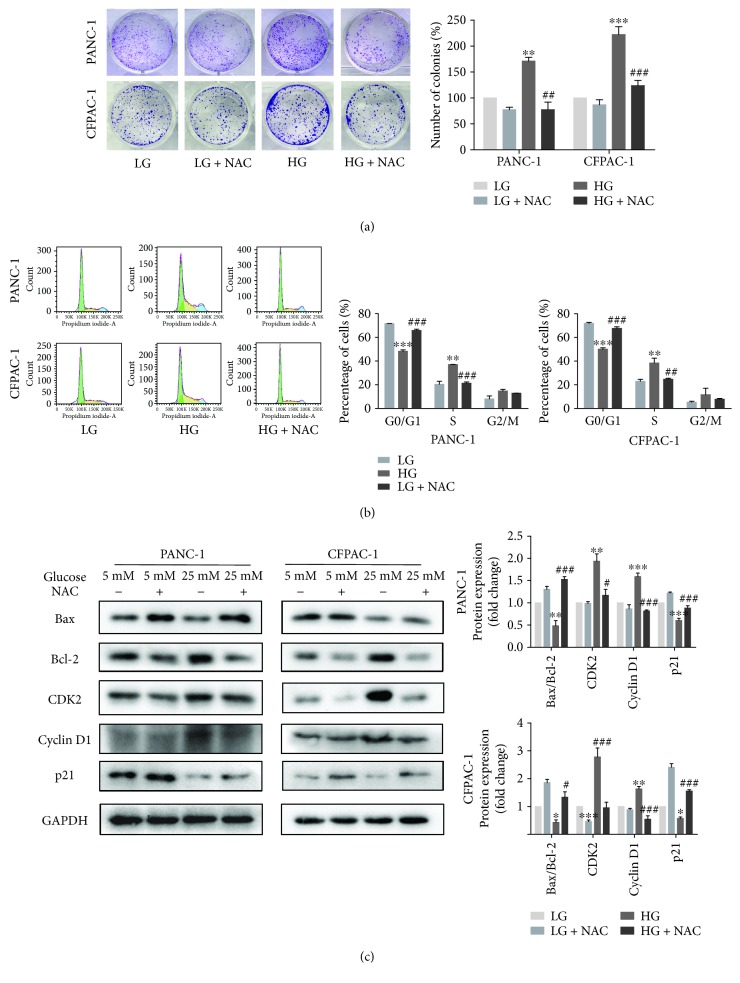
Suppression of ROS activation would reduce cell proliferation. (a) Colony formation assay following PANC-1 and CFPAC-1 cells incubated in LG and HG media in the presence or absence of 5 mM NAC. (b) Cell cycle distribution was performed by flow cytometric analysis. After stimulating with high glucose and NAC for 24 h, cells were collected and fixed with 70% ethanol for PI staining. The cell analysis was carried out using flow cytometry, and the results are shown as the percentage of cells in each phase of the cell cycle. (c) Cells were treated as described in (a), and then the proteins of Bax, Bcl-2, CDK2, cyclin D1, and p21 were detected by Western blotting. Data are presented as mean ± SEM (*n* = 3). ^∗^*p* < 0.05, ^∗∗^*p* < 0.01, and ^∗∗∗^*p* < 0.001, compared with the LG group. ^#^*p* < 0.05, ^##^*p* < 0.01, and ^###^*p* < 0.001, compared with the HG group.

ROS generation is associated with cancer cell proliferation [15, 24]. To verify whether the inhibition of ROS activation could suppress PC cell proliferation, after treatment with or without 5 mM NAC, colony formation assay showed that inhibiting HG-induced ROS generation resulted in a magnificent drop in colony formation (Figure 3(a)). Then, we explored the influence of NAC on the phase of the cell cycle for PC cells using flow cytometry after staining with PI. As shown in Figure 3(b), high glucose stimulated the progression of the G1 to S phase to a greater extent, compared with low glucose. After 24 hours of NAC treatment, the G1 phase rapidly accumulated from 47.1% to 66.9% (48.8% to 69.0%), and there was a corresponding reduction in the percentage of cells in the S phase (from 37.2% to 20.2%, 34.2% to 23.0%) in comparison with the high glucose-cultured cells. It indicated that treatment with NAC induced cell cycle arrest. Moreover, Western blotting assays were detected that the expression of cyclin D1 and CDK2 decreased, while the ratio of Bax/Bcl-2 and the expression of p21 enhanced in the HG and NAC treatment groups, compared with the HG group (Figure 3(b)). These results all illustrated that the inhibition of ROS activation will reduce cell proliferation.

### 3.4. The JNK Inhibitor SP600125 Promotes the Proliferation of HG-Induced Pancreatic Cancer Cells

**Figure 4:**
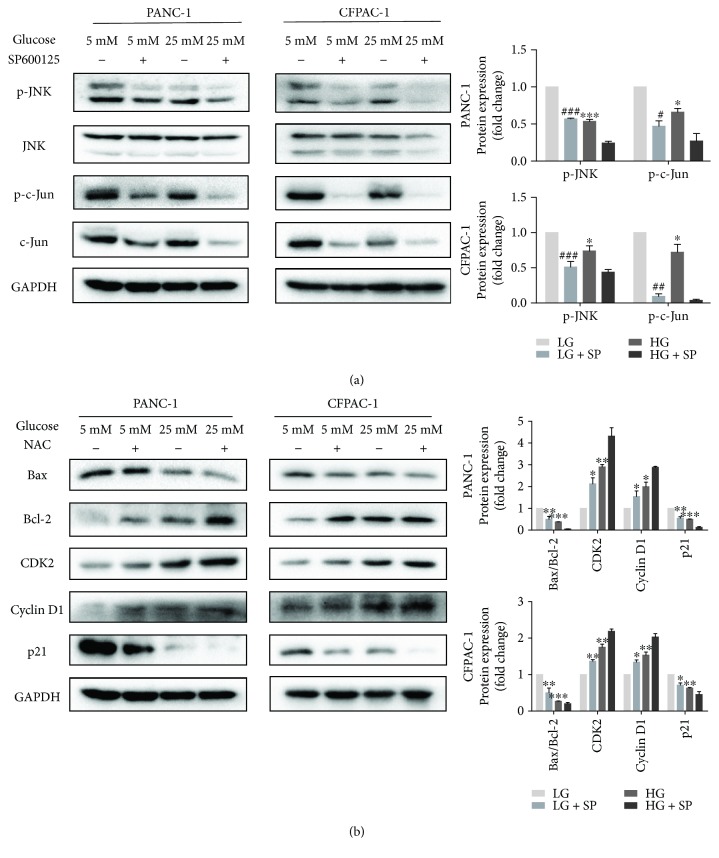
The JNK inhibitor SP600125 promotes the proliferation of high glucose-induced pancreatic cancer cells. (a) PANC-1 and CFPAC-1 cells cultured in LG and HG media for 24 h in the presence or absence of SP600125 (10 *μ*M). Cells were subjected to immunoblot analyses for the expression of p-JNK, JNK, p-c-Jun, c-Jun, and GAPDH. (b) Western blotting analysis of Bax, Bcl-2, CDK2, cyclin D1, and p21 in PANC-1 and CFPAC-1 cells after treatment as described in (a). Data are presented as mean ± SEM (*n* = 3). ^∗^*p* < 0.05, ^∗∗^*p* < 0.01, and ^∗∗∗^*p* < 0.001, compared with the LG group.

Primarily, JNK was deemed to mediate cell apoptosis in response to various stress signals [25, 26]. However, emerging evidence suggests that JNK plays a critical role in cell growth and survival in tumor cells [27, 28]. In order to investigate whether high glucose induced proliferation via inactivating phosphorylation of JNK and c-Jun, we treated PC cells with the JNK inhibitor SP600125 (10 *μ*M). Results showed that high glucose has the same effect as SP600125 which effectively inhibited the expression of p-JNK and p-c-Jun in PC cells (Figure 4(a)). In addition, after SP600125 treatment, the expression of cyclin D1 and CDK2 increased, and the ratio of Bax/Bcl-2 and the expression of p21 decreased (Figure 4(b)). Therefore, it can be proved that inactivation of JNK-mediated signaling has a pivotal role on HG-induced cell proliferation.

## 4. Discussion

Pancreatic ductal adenocarcinoma (PDAC) is one of the theriomas in the world with a rising incidence and is predicted to surpass breast, prostate, and colorectal cancers to become the second leading cause of cancer-related death by 2030 [29, 30]. New data show that up to 80% of patients with pancreatic cancer are either hyperglycemic or diabetic [31]. An increasing number of epidemiologic studies have found that diabetes mellitus (DM) has been proved to be an independent predictor of mortality from cancer of the pancreas [11, 32, 33]. In a pancreatic tumor microenvironment, hyperglycemia not only simply serves as the primary fuel for cells in the body, including of the brain and muscles, but also has a positive effect on the biological behavior of cancer cells [34, 35]. High glucose can promote pancreatic cancer cell proliferation, invasion, and metastasis [11, 36]. In the present study, our data certainly indicates that hyperglycemia promotes the proliferation of pancreatic cancer cells.

Cancer cell survival and growth are closely associated with tumor metabolism [37]. High cellular glucose metabolism has been considered as a hallmark of cancer [38]. Pancreatic cancer cells especially are highly active metabolically and programmed for enhanced nutrient uptake to supply energetic and biosynthetic pathways. Increased glucose consumption of pancreatic cancer cells is acquired to support their rapid proliferation and metastasis across the body. High glucose can not only be considered as the main calorie resource but also elevate expression of genes which are crucial in maintaining transformative phenotypes of cancer cells [39]. Here, comparing low glucose and high glucose conditions, we analyzed the activities in cell cycle regulation, cell proliferation and growth inhibition or apoptosis, and signal transduction. In high glucose conditions, we found upregulation of antiapoptotic gene Bcl-2 expression and downregulation of proapoptotic gene Bax expression; thereby, the changes demonstrate that high glucose inhibited apoptosis of pancreatic cancer cells. Meanwhile, the expression of cell cycle regulators like CDK2, cyclin D1, p21, and the cellular proliferation marker Ki67 also revealed that high glucose was able to promote cell proliferation. In addition, we also found that high glucose really increases ROS levels but inactivates the JNK pathway.

During the past decades, changes in cellular reactive oxygen species have long been implicated in tumorigenesis of the pancreas. As critical intracellular signaling molecules, the part where ROS plays as a tumor-promoting or a tumor-suppressing agent remains controversial. In fact, excess ROS production can induce damage in pancreatic cancer cells and induce apoptosis or necroptosis if not counteracted by antioxidant systems [24, 40, 41], whereas a moderate increase in ROS may have a mitogenic effect in tumors and promote pancreatic cancer cell progression [24]. Therefore, as downstream events, cell proliferation and growth inhibition or apoptosis depend on ROS levels. Similarly, researches have shown that ROS-inducing anticancer agents such as bardoxolone methyl, curcumin, and piperlongumine work through specific pathways and genes in pancreatic and other cancer cell lines and their effects have been linked to activation of high levels of ROS which are required for cancer chemotherapy [42–44]. Our experiment discovers that under high glucose conditions, ROS generation increases significantly and promotes the proliferation of pancreatic cancer cells. NAC, a ROS scavenging agent, inhibits the tumor-promoting effects of high ROS levels on pancreatic cancer cells and causes changes in cell cycle regulation. The cell cycle arrest is considered as a critical control point for the management of cancer cell growth [45]. Our results revealed that NAC could induce the G1/S-phase arrest to inhibit PC cell proliferation. Western blot analysis shows a substantial reduction in the expression of cyclin D1 and CDK2, and simultaneously an activation of p21 expression which are essential markers of cell cycle arrest. In addition, acting as a signal transduction messenger, ROS is reported with multiple functions including activation of c-Jun-N-terminal kinase (JNK). Many studies demonstrate that ROS can activate JNK, and the activation of JNK levels can be inhibited by ROS inhibitor NAC [46]. However, in our studies, ROS is involved in the regulation of antiapoptosis and promotes the proliferation of pancreatic cancer cells while JNK activity is inhibited. We found that the activity of JNK decreased in a time-dependent manner under high glucose conditions. When treated with the antioxidant NAC, the production of ROS is strongly blocked and the phosphorylation of JNK and c-Jun increases significantly.

Being one among the mitogen-activated protein kinase (MAPK) family, the c-Jun N-terminal kinase (JNK) is activated in response to various extracellular stimuli, such as genotoxic or cytotoxic stress, growth factors, and inflammatory cytokines [46]. It has been reported that the JNK signaling pathway regulates many key biological processes, including inflammation, apoptosis, cell survival, and cell proliferation, which function aberrantly in cancer [20]. Activation of JNK has been proposed to have critical roles in tumor development involve pro- and antitumorigenic functions. For example, short-term activation of JNK may increase the viability and growth of pancreatic cancer cells [25, 26, 47], whereas long period of activation may result in cell apoptosis such as through the E3 ubiquitin ligase ITCH-induced c-FLIPL turnover [24, 27, 28, 48]. JNK is mainly localized in the cytoplasm. When activated, partially activated JNK translocating into the nucleus activates intranuclear transcription factors, such as c-Jun, ATF2, and p53, by phosphorylation [28–30], thus enhancing the expression of downstream apoptosis-related genes and promoting apoptosis [20, 49]. In this study, our results reveal that treatment with high glucose resulted in a marked reduction of p-JNK. As it is known that p-c-Jun is the activated form of c-Jun [50], high glucose can also influence the expression of c-Jun itself and the phosphorylation of c-Jun. When PC cells are treated with the specific JNK inhibitor SP600125, consistent with our previous researches [51], we found that cell proliferation increases and apoptosis could be suppressed via downregulating the expression of p-JNK and p-c-Jun. SP600125 reduces the ratio of Bax/Bcl-2, inhibits the expression of p21, and promotes cell cycle progression. Thus, the present study indicates that the inactivation of JNK expression could contribute to cell proliferation.

In conclusion, the abovementioned findings demonstrate that ROS is required for high glucose-induced proliferation and cell cycle progression of pancreatic cancer cells. Moreover, the inactivation of the JNK pathway caused by the increase in ROS levels also has a pivotal role on HG-induced cell proliferation. These findings indicated that ROS stimulates proliferation of pancreatic cancer cells under high glucose conditions via inactivating the JNK pathway.

## Data Availability

The data used to support the findings of this study are available from the corresponding author upon request.
